# Spherical Silica Functionalized by 2-Naphthalene Methanol Luminophores as a Phosphorescence Sensor

**DOI:** 10.3390/ijms222413289

**Published:** 2021-12-10

**Authors:** Magdalena Laskowska, Anna Nowak, Mateusz Dulski, Peter Weigl, Thomas Blochowicz, Łukasz Laskowski

**Affiliations:** 1 Institute of Nuclear Physics Polish Academy of Sciences, PL-31342 Krakow, Poland; anna.nowak@ifj.edu.pl (A.N.); lukasz.laskowski@ifj.edu.pl (Ł.L.); 2 Faculty of Science and Technology, Institute of Materials Engineering, University of Silesia and Silesian Center for Education and Interdisciplinary Research, 75 Pulku Piechoty 1A, PL-41500 Chorzow, Poland; mateusz.dulski@smcebi.edu.pl; 3 Institute for Condensed Matter Physics, Technical University of Darmstadt, 64289 Darmstadt, Germany; Peter.Weigl@physik.tu-darmstadt.de (P.W.); thomas.blochowicz@physik.tu-darmstadt.de (T.B.); 4 Institute for Applied Physics, Technical University of Darmstadt, 64289 Darmstadt, Germany

**Keywords:** 2-naphthalenemethanol, phosphorescence, triplet state solvation dynamics, nanocomposite

## Abstract

Photoluminescence is known to have huge potential for applications in studying biological systems. In that respect, phosphorescent dye molecules open the possibility to study the local slow solvent dynamics close to hard and soft surfaces and interfaces using the triplet state (TSD: triplet state solvation dynamics). However, for that purpose, probe molecules with efficient phosphorescence features are required with a fixed location on the surface. In this article, a potential TSD probe is presented in the form of a nanocomposite: we synthesize spherical silica particles with 2-naphthalene methanol molecules attached to the surface with a predefined surface density. The synthesis procedure is described in detail, and the obtained materials are characterized employing transmission electron microscopy imaging, Raman, and X-ray photoelectron spectroscopy. Finally, TSD experiments are carried out in order to confirm the phosphorescence properties of the obtained materials and the route to develop phosphorescent sensors at silica surfaces based on the presented results is discussed.

## 1. Introduction

Since the last decades of the 20th century, organic materials have shown a vast potential for application. One of the significant properties of such compounds is photoluminescence. This phenomenon, especially one of its types, fluorescence, is most commonly used to study biological systems. However, the decay time for such materials is faster than the one of, for example, phosphorescence. The latter gives a longer excitation time and has the potential for application in imaging, monitoring, or even sensing. Mukherjee and Thilagar showed that well-known organic molecules could be designed using old and new concepts to obtain new products with efficient phosphorescence features [1]. These approaches can use hydrogen or halogen bonding and heavy atom effects. Hydrogen bonding plays a crucial role in biological structure. It is known that, in the biomolecules of living cells, it is responsible for stability, structure, and functionality [2]. The popular approach to enhance phosphorescence at room temperature is to connect organic compounds, such as boron-dominate [3] or naphthalene and its derivates [4], with amorphous polymer matrices, such as polymethyl methacrylate [5], polyvinyl alcohols [6], or cellulose acetate [1]. Naphthalene (Nph) and its derivates, as one of the simplest polyaromatic hydrocarbons, consist of two fused benzene rings, and are commonly used as fluorescence markers and are considered as sensors for metal ions. Still, their potential for applications is much broader: for example, as a component for dyes, drugs, or surfactants, just to name of few [7]. Nph is transparent, biocompatible, hydrothermally stable, and can immobilize organic functional groups in its framework, giving more ways to investigate biological systems. In addition, Nph also provides the opportunity to perform local solvation experiments using phosphorescence, so-called triplet state solvation dynamics (TSD) [8]. For such TSD experiments, Nph (or another suitable solute) is dissolved and highly diluted as a TSD probe (phosphorophore) in the solvent under investigation. The resulting thermodynamic equilibrium consisting of the TSD probe and the surrounding solvent can be locally disturbed by exciting the phosphorophore into a metastable triplet state by a UV laser pulse via an intersystem crossing. This initiates reorientation dynamics of the solvent molecules in the first solvation shell, i.e., approximately in a single molecular layer of solvent molecules (∼1 nm), resulting in a spectral shift of the phosphorescence emission as a function of time. Depending on the TSD probe used, local dielectric or shear mechanical experiments can be performed; thus, allowing the TSD method to be understood as a local version of dielectric or shear relaxation spectroscopy, respectively [8,9,10].

Based on these findings, the advantages of the TSD come into play when it is difficult to draw conclusions about microscopic properties using macroscopic measurement methods. For the dielectric spectroscopy, this applies, e.g., if cross-correlation effects become relevant in the investigated sample as illustrated in a recent publication [11] or if the measurement itself is influenced by the Maxwell–Wagner polarization effect as in porous systems, which are sometimes used as a simplified model for biological cells [12]. In a recent study on biological cells, dielectric spectroscopy is used to discern the macroscopic electrical properties of a multitude of biological cell suspensions and cell tissue types and reveal information regarding time-dependent cellular movement—e.g., cell sedimentation [13]. The TSD as local dielectric spectroscopy can give more important information about the basic properties of a biological system. Therefore, the search for a universal TSD probe seems to be an interesting issue but still not explored on a large scale.

In order to fully exploit the local character of TSD, and thus the full potential of this method, TSD probes are needed that still function as expected when covalently bound to a surface [14]. Although some experience was gained in this regard in previous studies [2,14,15], it is, unfortunately, not yet possible to completely predict the behavior of possible TSD probes as such. Instead, the suitability must be tested on a case-by-case basis. However, if the exact location of the TSD probe is known due to covalent bonding, the dynamic information obtained by the TSD method is not only local but can be unambiguously assigned to a certain local environment, which opens up completely new fields of application for the method. In this way, it is conceivable, for example, to resolve interfacial effects at surfaces by connecting TSD probes at different distances from the surface, and thus to investigate questions that are difficult or impossible to access with macroscopic measurement techniques [14].

To take a step in this direction, it is reasonable—based on the results of ref. [2]—to functionalize surfaces with Nph or its derivatives and subsequently test them with regard to their applicability for TSD investigations. For this purpose, the probe molecules must be properly separated on the surface and screened from each other in order to facilitate their behavior as TSD probes. A promising solution can be the separation of naphthalene (or its derivatives) on proper support by controlling its distribution according to the solid solvent concept [16]. The ideal matrix for such a separation seems to be a spherical nano-silica. This material, being in the form of 300 nm spheres, allows for the trapping of naphthalene methanol (NphMet) at its surface via propyl-carboxyl units. In order to keep the intended distances between active molecules, spacer units were used [17]. Moreover, due to the spherical shape, different molecules of NphMet on the surface of the same particle are screened from each other to a certain extent.

**Figure 1:**
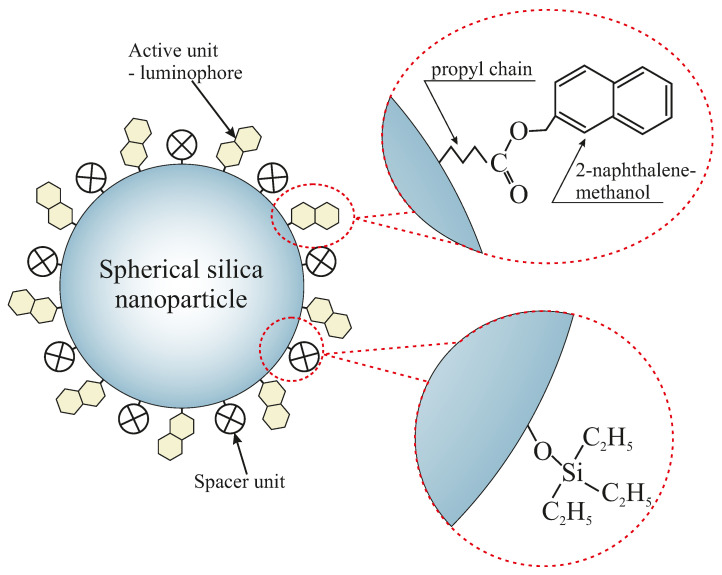
The scheme of a spherical silica functionalized by 2-naphthalene methanol with designed inter-molecular distances. The spacer units play a role as separators between functional molecules.

This paper presents a method of obtaining 2-naphthalene methanol molecules attached to the amorphous spherical nanosilica surface, as presented in Figure 1.

We investigated the material with regards to the concentration of the active molecules. For this reason, we prepared samples containing different concentrations of 2-naphthalene methanol molecules at the spherical silica surface. The concentration of functional molecules, and thus the distance between them, was set by the use of functional units. More precisely, we modified the distribution by a variation of the ratio between 2-naphthalene methanol units and spacer molecules—triethylsilane in this case. The higher the number of spacer units per single functionality, the more separated the functional molecules are from each other. Each functional unit was attached to the silica’s surface via a propyl-carboxylate molecule. For this investigation, we choose 1, 6, and 15 spacers per functional unit. Thus, we named samples consequently:

- Sil-S-COONph 1—spherical silica functionalized with 2-naphthalene methanol, containing one spacer unit per single functional group
- Sil-S-COONph 6—spherical silica functionalized with 2-naphthalene methanol, containing six spacer units per single functional group
- Sil-S-COONph 15—spherical silica functionalized with 2-naphthalene methanol, containing 15 spacer units per single functional group

As references, we measured samples containing exclusively anchoring units (carboxylic acid) and spacer units in proper concentrations. Samples were denoted as follows:

- Sil-S-COOH 1—spherical silica functionalized with propyl carboxylic acid molecules, containing one spacer unit per single functional group
- Sil-S-COOH 6—spherical silica functionalized with propyl carboxylic acid molecules, containing six spacer units per single functional group
- Sil-S-COOH 15—spherical silica functionalized with propyl carboxylic acid molecules, containing 15 spacer units per single functional group.

All the samples were prepared in the form of a powder.

## 2. Materials and Methods

### 2.1. Synthesis of Silica Nanoparticles Functionalized with 2-Naphthalene Methanol

First, the spherical silica nanoparticles were prepared according to the optimized Stöber procedure [18]. The procedure is based on the hydrolysis and polycondensation of tetraethyl orthosilicate (TEOS—Sigma-Aldrich Chemie GmbH, Steinheim am Albuch, Germany). The synthesis results in homogeneous silica spheres with a diameter of 300 nm (the analysis of the diameters of silica spheres showed 299.8 ± 11.4 nm—see: Supplementary Materials).

**Figure 2:**
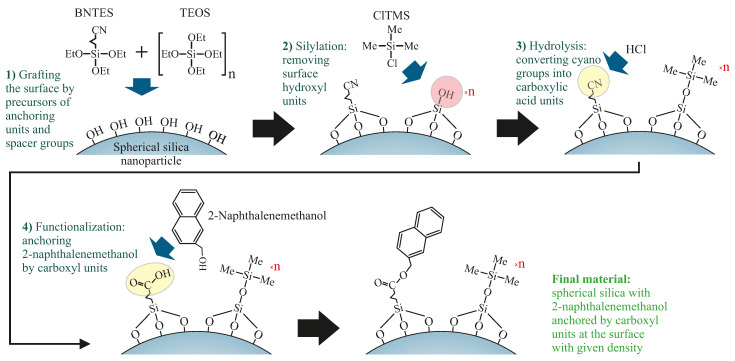
A diagram of the route of the synthesis of the spherical silica functionalized by 2-naphthalene methanol with a designed density of functional units. The number n denotes the number of tetraethyl orthosilicate (TEOS) in moles per single mole of the precursor of functional units (butyronitriletriethoxysilane—BNTES) and determines the density. Other abbreviations: TEOS—tetraethyl orthosilicate, ClTMS—chlorotrimethylsilane, Me—methyl groups, Et—ethyl units.

Next, the as-synthesized spherical silica were functionalized by 2-naphthalene methanol with a given density at the surface, according to steps presented in Figure 2.

All the synthesis details are described in the Supplementary Materials.

### 2.2. Characterization Methods

X-ray photoelectron spectroscopy (XPS) measurements were realized with the use of a Physical Electronics PHI 5700 spectrometer using monochromatic Al Kα radiation. The photoelectrons were collected at an angle of 45°. The survey spectrum showed the presence of principal core level lines from Si, O, and C with no evidence of impurities. The high-resolution XPS spectra for all core levels were corrected for the background signal using the iterated Shirley algorithm, while the bands were curve-fitted by a composition of Gaussian and Lorentzian lines in the Multipack software.

The sample morphologies were confirmed by Transmission Electron Microscopy (TEM) with an FEI Tecnai G2 20 X-TWIN equipped with a LaB${}_{6}$ emission source and an FEI Eagle 2 K CCD camera.

A WITec confocal Raman microscope CRM alpha 300R equipped with an air-cooled solid-state laser (λ = 532 nm) and a CCD camera was used for structural characterization. The excitation laser radiation was coupled into the microscope through a single-mode optical fiber with a 50 μm diameter. An air Olympus MPLAN (100×/0.90NA) objective was used. Raman scattered light was focused on multi-mode optical fiber (100 μm diameter) and a monochromator with a 600 lines/mm grating. Raman spectra were accumulated using 20 scans with an integration time of 20 s and a resolution of 3 cm${}^{-1}$. The monochromator was calibrated using the Raman line of a silicon plate (520.7 cm${}^{-1}$). The baseline correction and cosmic ray removal were conducted using the WitecProject Plus 4 software. Taking into account the uniformity of the core material, the Raman spectra of reference and 2-naphthalene methanol-functionalized samples were normalized to the silica band (457 cm${}^{-1}$). Finally, the peak fitting analysis was performed on such prepared data using the GRAMS 9.2 software package.

The triplet state solvation dynamics (TSD) experiment is described elsewhere in detail [2,9,19]. In a nutshell: the sample under investigation, i.e., functionalized spherical silica in 1-propanol (99.9% purity from Alfa Aesar) is filled in a rectangular quartz glass cell, which is mounted in an optical cryostat (Conti Spectro 4 from CryoVac). The excitation of Nph-based TSD probe molecules is performed by UV laser pulses of wavelength 266 nm with ∼2 mJ pulse energy. These are provided by a pulsed 10 Hz laser system with an integrated pulse divider, which is based on a Spitlight 600 Nd:YAG laser from Innolas. For the measurements shown here, the repetition rate was set to $f_{laser}=0.1$ Hz, so that $f_{laser}\leq 1/\left(3\;\tau_{probe}\right)$ is satisfied since for Nph-based probe molecules, the phosphorescence lifetime is $\tau_{probe}\leq 3\;$ s [2]. The emitted phosphorescence of the TSD probe molecules is collected under $90^{{}^{\circ}}$ of the incident laser beam and guided through a liquid light guide fiber to the detection module. The latter consists of a Czerny–Turner grating spectrograph (Shamrock 500i from Andor Technology) and an iCCD camera (iStar 340T, also from Andor Technology) with an integrated gate and delay generator. To obtain the phosphorescence spectra presented below, the emitted phosphorescence was dispersed using a 150 lines/mm grating, while the time resolution was set to Δt/t=10%.

## 3. Results and Discussion

### 3.1. Transmission Electron Microscopy Imaging

**Figure 3:**
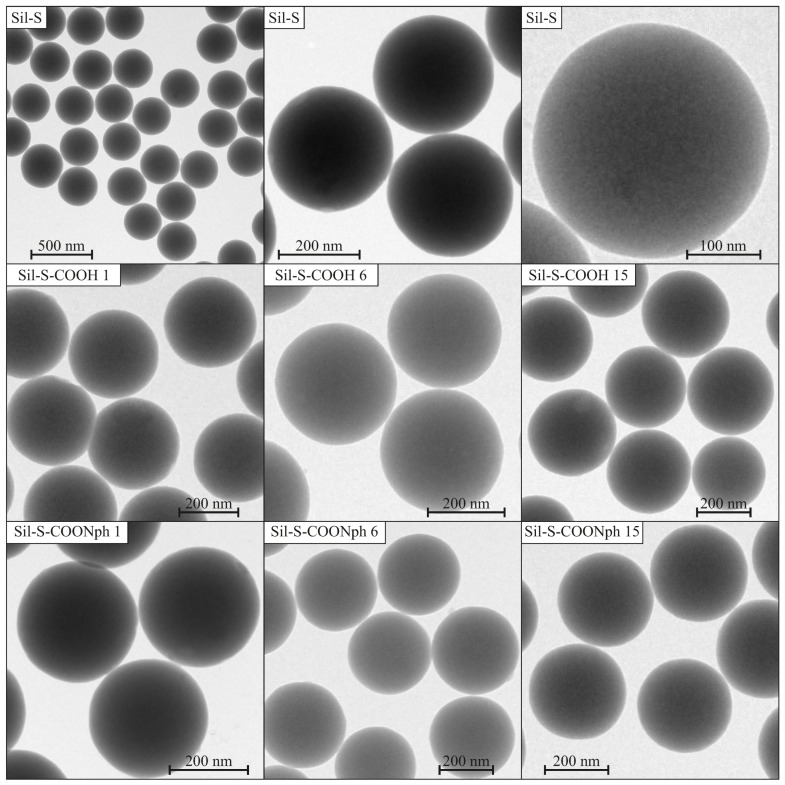
TEM images of functionalized samples. Samples containing only anchoring units (**middle row**) and those containing 2-naphthalene methanol (**bottom row**) are juxtaposed with the images of non-functionalized spherical silica (**upper row**).

In order to check the purity of the samples and observe the surface of functionalized materials, we carried out TEM imaging. The TEM images of investigated samples (both 2-naphthalene methanol-containing samples and those with just anchoring units) together with pure spherical silica (denoted as Sil-S) are presented in Figure 3.

The reference material—spherical silica before functionalization—shows the correct structure: the spheres are homogeneous with a diameter of 300 nm, according to our assumption (Figure 3—upper row). Neither impurities nor structural defects are visible. For this reason, we can assume that synthesis was started from the correct substrate material.

After functionalization, the structure did not visibly change (Figure 3—middle and bottom rows), i.e., the shape and diameter of the spheres is unaltered. Neither impurities can be seen nor agglomeration of particles. On this basis, we can conclude that in the case of 2-naphthalene methanol being present on the surface (see section further below), only molecules are attached to the surface, instead of bulk material, which would be clearly visible under TEM.

### 3.2. X-ray Photoelectron Spectroscopy Measurements

**Figure 4:**
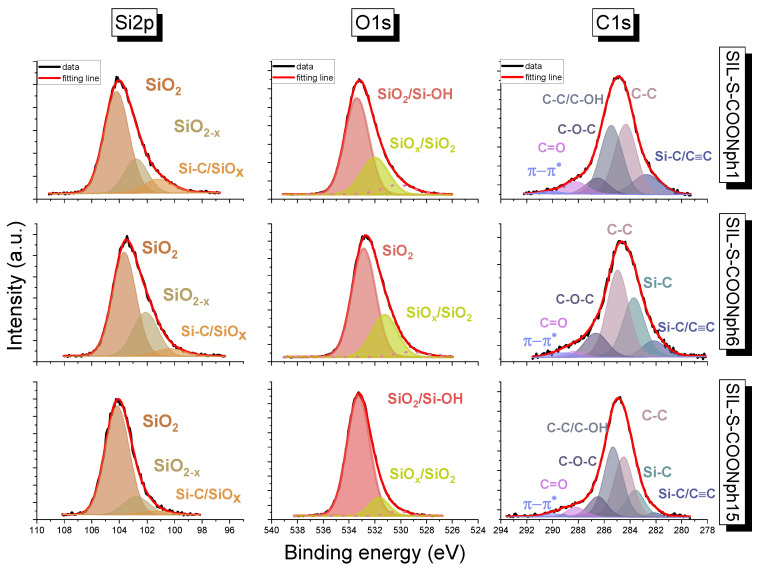
Deconvoluted high-resolution Si2p, O1s, and C1s X-ray photoelectron spectra of the 2-naphthalene methanol-containing samples.

**Figure 5:**
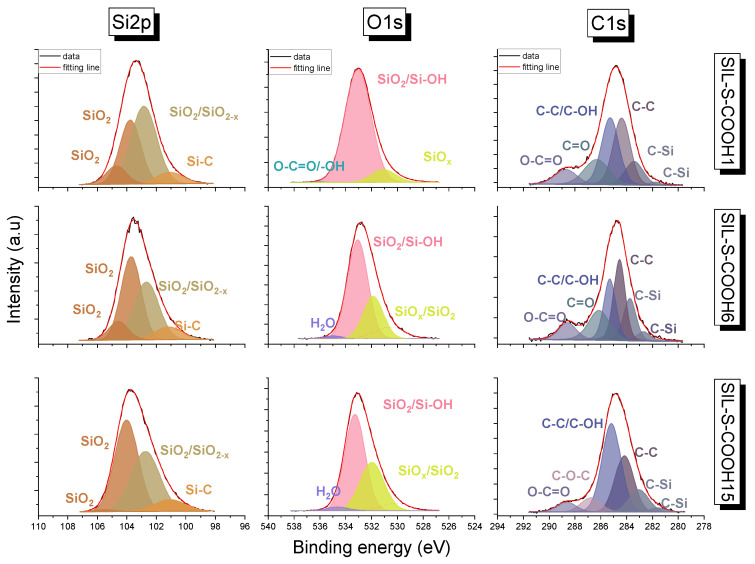
Deconvoluted high-resolution Si2p, O1s, and C1s X-ray photoelectron spectra of the carboxylic acid-containing samples.

To confirm surface silica functionalization, X-ray photoelectron spectroscopy (XPS) measurements were performed. The experiment indicated chemical bonding on the surface of the silica samples. Two series of samples were compared: one containing the proper functional units (2-naphthalene methanol) and another containing anchoring units (carboxyl groups) as a reference. High-resolution core-level spectra of Si2p, O1s, and C1s are presented in Figure 4 and Figure 5, whereas element compositions of the surface are summarized in Table 1 and Table 2.

The Si2p spectrum for samples with -COOH groups was deconvoluted into four peaks, while three resulted for the 2-naphthalene methanol-containing surface. In all samples, peaks with low binding energy (from 100.3 to 101.2 eV) were ascribed to Si–C bonding or SiO${}_{x}$ [20]. However, the low energy state can be caused by charge neutralization via flood guns. In Figure 4 and Figure 5, the most intensive peaks for the Si2p line are derived from SiO${}_{2}$ [21]. The smaller peaks with binding energy in the range from 102.1 to 102.8 eV were attributed as SiO${}_{2-x}$. The C1s line for COOH-containing samples revealed the presence of six peaks. The main peak at 284.4, 284.6 and 284.2 and 285.3 eV for Sil-S-COOH 1, Sil-S-COOH 6, and Sil-S-COOH 15, respectively, can be associated with the C-C bond and oxidized alkane-type carbon atoms (C-C/C-OH) or bonding between C-Si-O. The other carbon peak in the Sil-S-COOH 1 material is located at 286.3 eV. It is derived from C=O. The peak at 286.2 eV can be associated with the C-O bond in the Sil-S-COOH 6 sample. The highest peak with binding energy at 286.8eV was attributed to the C-O-C bond in the last sample of the series. The peaks at 288.65 eV are related to the O-C=O band in all Sil-S-COOH samples. In both series of samples, the smallest peak at the lowest binding energy, around 282.2 eV, arises from C-Si [21]. However, in the 2-naphthalene methanol-containing samples, the same binding energy value can be ascribed to C≡C. Ruangchuay et al. [22] also reported that the main C-C peak at around 284.5 eV arises from a fused pair of benzene rings. The small peaks at the lower binding energy of 283.7 eV for a small amount of naphthalene on the surface (samples Sil-S-COONph 6 and Sil-S-COONph 15) ascribed to Si-C can also be derived from naphthalene [21]. The C1s of the 2-naphthalene methanol-containing samples revealed a signal consisting of C-C or C-OH bonds located at 285.4 eV, the same as in -COOH-containing samples. The peaks at 286.6 eV in all sample series can be attributed to C-O-C. The other carbon peak at 288.2 eV was derived from C=O (Figure 4). In 2-naphthalene methanol-containing samples, carbon π−π* interaction at about 290.2 eV was observed. This shake-up satellite peak confirmed the presence of aromatic carbon derived from naphthalene [22]. The O1s resolved lines for both sample series were related to tge contributions of the different components. The highest peaks correspond to SiO${}_{2}$ and Si-OH (from 533.1 to 533.4 eV) [23]. By contrast, the smallest peak corresponds to SiO${}_{x}$ or SiO${}_{2}$ in all samples with relative intensities at 531.1 eV and shifts to the side of higher energies by 1.2 eV. It suggests the change in the oxidation state of non- stochiometric silica oxide. Furthermore, at the surface of -COOH-containing samples, chemisorbed OH groups are observed, resulting from small amounts of water contamination [23].

### 3.3. Raman Spectroscopy

**Figure 6:**
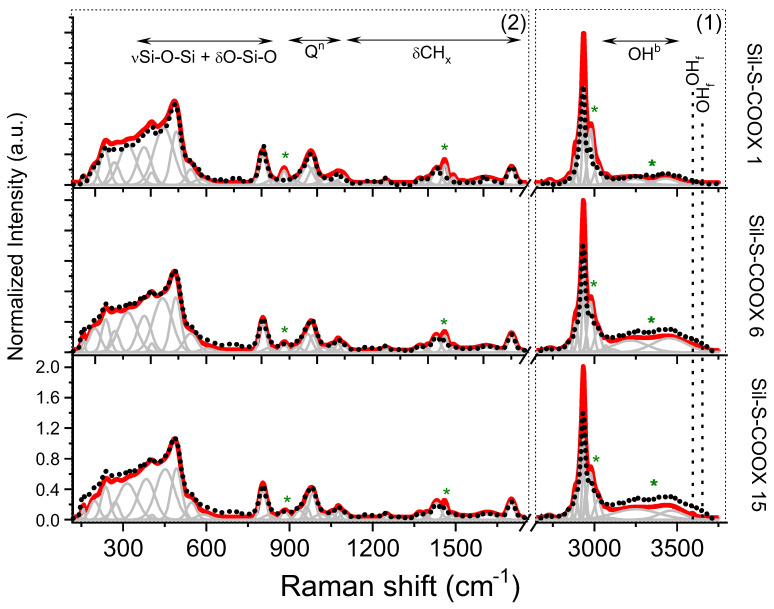
Raman spectra of Sil-S-COOX samples with different concentrations of active units, while X = H or 2-naphthalene methanol. Spectra of reference samples (not containing 2-naphthalene methanol) are presented as dotted lines, while 2-naphthalene methanol-activated silica as continuous red lines. Raman spectra were normalized to better illustrate the differences between the reference and activated samples. Additionally, all spectra were fitted using the Voigt function with the preservation of a minimum number of components. Green stars indicate spectral modifications after the silica functionalization through the 2-naphthalene methanol.

Samples with different concentrations of propyl-carbonate units (-COOH) and 2-naphthalene methanol (NphMet) were subjected to more detailed structural analyses by Raman Spectroscopy (RS). The spectra of reference samples (COOH-containing samples) were compared with their 2-naphthalene methanol-functionalized counterparts (Figure 6).

According to the data, the spectra of propyl-carboxylic acid-functionalized silica (dotted-highlighted spectra) were divided into two spectral regions: (1) 3800–2750 and (2) 1850–120 cm${}^{-1}$ (Figure 6). The first region corresponds to the stretching vibration of hydroxyl groups, as well as the symmetric and asymmetric stretching modes of CH${}_{x}$ (*x* = 2, 3) groups. The latter region includes (i) stretching vibration of carbonyl C=O, alkane C-C and deformation of CH${}_{x}$ groups within propyl-carbonate chains and silyl fragments (1800–1250 cm${}^{-1}$), (ii) silicon-oxygen tetrahedral modes (1250–850 cm${}^{-1}$) overlapping with deformational modes of propyl-carbonate moieties (1350–800 cm${}^{-1}$), as well as, (iii) the medium-range order silica vibrations corresponding to lattice defects (850–150 cm${}^{-1}$) [24,25]. Moreover, the anchoring of 2-naphthalene methanol into the propyl-carbonate molecular fragment generally modifies the band’s intensity in region (1). In turn, the presence of single 2-naphthalene methanol molecules can also be followed indirectly through structural alteration within the silica network and the propyl-carbonate chain, as evidenced in region (2).

A more precise analysis of the reference data and their comparison with the spectra of the functionalized samples in region (1) revealed bands typically ascribed to hydroxyl groups, i.e., water molecules, or H-bonded patterns (3800–3200 cm${}^{-1}$). Some explanation is related to the different content of the -COOH molecular fragments anchored to the propyl chain. In turn, a relatively large full width at half maximum (FWHM) found for non-functionalzed systems may be related to the high dynamicity of the individual units (Figure 6). A similar situation appears regardless of the concentration of functional units anchored to the surface of the reference silica. Interestingly, the smaller content of propyl-carboxyl acid groups, the higher number of hydroxyl groups involved in the H-bonding scheme. A similar effect was observed for bands of the 3800–3600 cm${}^{-1}$ range that point to a variable number of the non-hydrogen-bonded or even free hydroxyl groups (OH${}^{f}$ in Figure 6) depending on the content of propyl-carbonate moieties. It seems that only some fraction of hydroxyl groups has the possibility to interact with each other and with lesser propyl-carbonate groups that have been anchored to the surface of the silica.

The addition of the 2-naphthalene methanol causes the Raman spectra in region (1) to show a slightly lower intensity than the hydroxyl-related bands of the other samples. Moreover, the trend observed upon changing the organic modifier content is similar to the one observed for the non-functionalized material, i.e., the lower content of 2-naphthalene methanol, the higher intensity of the hydroxyl-related bands is. This effect corresponds to weaker interactions within the H-bonding scheme. Furthermore, an intensity decrease of the bands related to the free hydroxyl groups observed in all three systems suggest the anchoring of the organic molecules to the propyl chains, and thus confirms that the synthesis procedure works as expected.

Other bands (3200–2750 cm${}^{-1}$) observed in the reference and 2-naphthalene methanol-functionalized samples are assigned to the modes of CH${}_{x}$ (*x* = 1, 2, 3) groups. A slight increase in the intensity of the bands within this part of the spectrum may confirm the presence of additional functional CH groups at aromatic rings of naphthalene (Figure 6), as previously mentioned.

At fingerprint region (2), a signal originated from the stretching and deformational modes of the silica network overlaps with the vibration of propyl chains (Figure 6). Unfortunately, there are no specific differences when comparing the -COOH-containing samples with a variable number of active groups. Looking more precisely, some differences result from the position and number of bands, while at least slight differences appear in the respective intensities. In this context, all three reference samples are characterized by a similar band arrangement with the main bands of the 1850–1250 cm${}^{-1}$ region assigned to the stretching vibration within the COOH fragment (∼1701 cm${}^{-1}$), as well as stretching C-C and deformation CH${}_{2}$ modes. Other bands found below 1250 cm${}^{-1}$ are related to the silica units. In the -COOH-containing samples, spectra are characterized by four bands at 1090, 1043, 988, and 960 cm${}^{-1}$, which suggest the formation of substructures with terminal oxygen atoms located within the Q${}_{3}$, Q${}_{4}$, and Q${}_{2}$ units, and indicate stronger structural disordering [26,27]. All bands may indicate the depolymerization of the silica network due to the synthesis and formation of non-bridging oxygen molecular fragments (NBO), which facilitate the anchoring of the propyl-carbonate [20,26]. Furthermore, the presence of the band at around 800 cm${}^{-1}$ may indicate Si vibration in an oxygen cage. In turn, the bands centered at low frequencies correspond mainly to the O-bending motion of n-membered silica rings with n = 3, 4, 5, 6…, where the location is related to the number of oxygens within the ring. Interestingly, the main bands ascribed to the individual type of ring structures are slightly shifted toward lower frequencies compared to the literature data. The modification of the structural silica network as a result of the synthesis was also observed. This may suggest the formation of defected structural fragments in the form of Si${}_{2}$O${}_{6}^{4-}$, Si${}_{2}$O${}_{5}^{2-}$, and SiO${}_{2}$, which confirms the presence of Q${}^{2}$, Q${}^{3}$, and Q${}^{4}$ units.

Some differences have appeared after the sample functionalization by 2-naphthalene methanol. Practically the whole band arrangement, including the band position, number, and intensity, remains unchanged after the introduction of 2-naphthalene methanol. Some structural alterations linked to the changes in the intensity of the bands were found only in the case of two bands located at 1463 and 883 cm${}^{-1}$. According to the data mentioned above, those bands are related to the propyl chain, while the modification of its intensity may correspond to molecular reorganization due to the external modifier. These observations are well correlated with the naphthalene concentration in the system, i.e., the lower the number of external organic groups, the lower the intensity of such bands and the spectrum looks as in the case of a non-functionalized system (Figure 6).

The juxtaposition of all the individual Raman models can be found in Supplementary Materials.

### 3.4. Triplet State Solvation Dynamics

The phosphorescence investigations on the functionalized spherical silica particles were carried out in 1-propanol. 1-propanol is a solvent whose local reorientation dynamics are already well understood using polar and nonpolar TSD probes, making it ideally suited to characterize the functionalized samples and thereby elucidate their applicability for local TSD experiments [9,10,15]. In previous characterizations [2,15], it has proven useful to perform investigations in the glassy state initially since this ensures that the solvent does not relax in the accessible measurement window and, therefore, the local reorientation dynamics of the solvent does not affect the spectrum of the probe molecule. The low glass transition temperature of 1-propanol $T_{g,cal}\approx 96$ K [28] and the resulting weak thermal line broadening represents another advantage for the characterizations of the functionalized particles regarding their capability as TSD probes.

**Figure 7:**
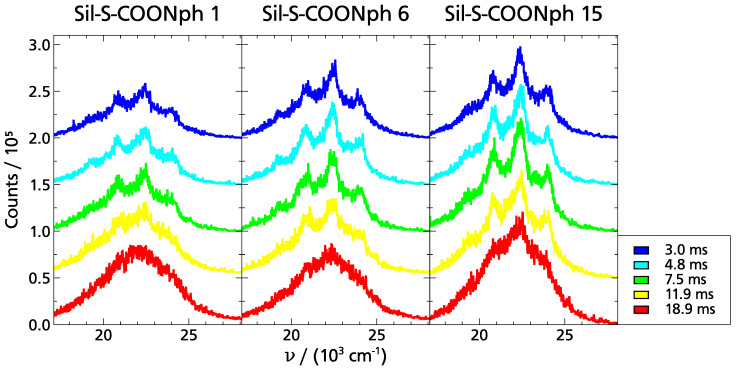
TSD measurement on functionalized spherical silica in 1-propanol at T=90 K. Phosphorescence spectra are shown for different times after excitation of the dye molecules by the UV laser pulse. For better clarity, the spectra are shifted in the *y*-direction at different delay times *t*. From left to right, the distance between different NphMet molecules anchored to the spherical silica particles increases. For all functionalizations, it can be observed that the visible structure in the spectra decreases as *t* increases.

**Figure 8:**
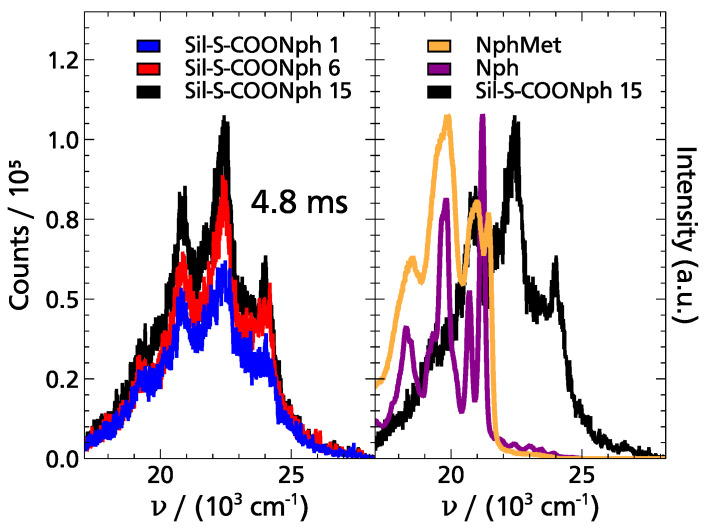
TSD results on functionalized spherical silica in 1-propanol at T=90 K. (**Left**) The comparison of phosphorescence spectra of the different functionalizations at t=4.8 ms reveals that the measured intensity increases with increasing distance between different NphMet groups. (**Right**) Comparison of the phosphorescence spectra of Sil-S-COONph 15 with the spectra of pure Nph, respectively, NphMet dissolved in 1-propanol. The latter spectra were recorded at t=200 ms. The maximum intensity of each spectrum was normalized. It can be observed that although all spectra look similar in their peak structure, the spectra of the functionalized samples are significantly shifted in energy (∼2600 cm${}^{-1}$).

For these reasons, the phosphorescence spectra of the three functionalized samples shown in Figure 7 were recorded in 1-propanol at T=90 K $<T_{g,cal}$. As can be seen from the figure, phosphorescence spectra were obtained in all three samples from 3 up to 18.9 ms. The structure of the spectra is similar for all functionalizations; only the intensity increases slightly with the increasing distance of the phosphorophoric moieties. The left part of Figure 8 further illustrates this observation. Nevertheless, the time range in which these spectra could be recorded is interesting, since, for the TSD probe, Nph, as well as its derivative’s typical phosphorescence lifetimes are $\tau_{phos.}>2$ s for $T\leq T_{g}$ [2,10,14]. Based on these data, one would have expected to observe a phosphorescence signal in the time range of 1 ms up to several seconds, i.e., in a range of up to three decades in time [2,10,14,15]. In contrast, in the data shown in Figure 7, at the longest delay times, the characteristic peaks of the spectra are hardly visible anymore. Instead, the signals at 18.9 ms rather show only the luminescence of 1-propanol [14]. Therefore, not only the phosphorescence lifetime is dramatically shortened in the functionalized samples, but also the signal intensity seems to be reduced. In addition to this, from the right part of Figure 8, the surprising observation can be made that the phosporescence spectra of the functionalized samples are significantly shifted, namely by ∼2600 cm${}^{-1}$ with respect to those recorded with Nph and NphMet in 1-propanol (The fact that the spectra of Nph and NphMet differ somewhat in the intensity of the different peaks is due to the concrete molecular structure of the TSD probe and is in general not relevant for TSD measurements since only the relative time-dependent shift of the entire spectrum is of interest).

Thus, it can be stated that the functionalized samples do not show the expected spectroscopic behavior from the TSD point of view. The reason for this surprising result cannot be found in the dynamics of the solvent used, since below the glass transition temperature $T_{g}$, 1-propanol is unrelaxated in the accessible time window. Therefore, the cause of these observations must be related to the phosphorophoric moieties, i.e., mainly the Nph group. From studies on triplet eximers (excited dimers), it is known that spectral changes, as well as the shortening of phosphorescence lifetimes, occur when the concentration of probe molecules (such as Nph) in solution is increased, which is equivalent to an increase in the probability of interaction between probe molecules and thus also of dimer formation [29,30,31]. Following this idea, the observations made here on the functionalized silica particles can be interpreted as the individual phosphorophoric moieties interacting with each other because their spatial separation is not large enough. The fact that the intensity increases slightly with increasing distance between the phosphorophoric moieties also supports this interpretation.

An alternative, although unlikely, approach to explain the observations is that the relevant TSD probe properties of Nph or NphMet are influenced by the functionalization process itself. At least the reduced phosphorescence lifetime is reminiscent of the so-called heavy atom effect [32]. If true, the connection to the carboxyl group would lead to such a significant shift in the electron configuration of the Nph that the observed effects occur. However, even if the slight spectral shift between pure Nph and pure NphMet dissolved in 1-propanol points in the same direction, it is rather unlikely that a spectral shift in the order of ∼2600 cm${}^{-1}$ can be explained in that way.

Regardless of the exact explanation of the observations, a three-step strategy emerges from the presented results to be able to produce functionalized silica surfaces that allow for time-resolved phosphorescence solvation measurements in the future. First, the distance between each two phosphorophoric moieties must be further increased. This can be done, on the one hand, by increasing the ratio of spacer units to phosphorophoric moieties. At the same time, increasing the diameter of the spherical silica particles can also be considered. Moreover, it is conceivable to use higher Nph-alcohols instead of NphMet in the second step in order to increase the average distance from the phosphorophoric center to the anchoring point. Furthermore, as a last resort, it is also possible to vary the TSD probe itself. For example, functionalizations based on quinoxaline or indole instead of Nph could be considered. All of this will be the subject of future work.

## 4. Conclusions

In the present paper, we demonstrate a route to synthesize functionalized spherical silica particles with phosphorescent dye molecules of a defined surface density with the long term aim to use the phosphorophoric centers as sensors to locally probe slow solvent dynamics close to surfaces and in interfacial layers via the triplet state solvation dynamics technique. As a first step, the functionalization of spherical silica with 2-naphthalene methanol was performed for three different densities of the chromophore. The resulting functionalization was characterized in detail, and it was shown that we indeed obtained the materials with the intended molecular structure: while the structure of the spherical silica particles is preserved, 2-naphthalene methanol molecules are attached to the surface via propyl-carbonate groups with the predefined concentrations.

Finally, phosphorescence spectra were recorded at various delay times and a dramatically shortened phosphorescence lifetime and a reduction of intensity along with a huge wavenumber shift were observed compared to dissolved pure probe molecules in the same solvent. However, based on the present study, the route is open to realize smaller surface densities and increase the distance between phosphorophoric centers and the anchoring points. It may reduce possible interactions between chromophores and also with the silica surface. In that way, the potential of phosphorescent sensors at silica surfaces is expected to be accessible in the near future.

## Figures and Tables

**Table 1:** Surface elemental composition of 2-naphthalene methanol-containing samples determined by X-ray photoelectron spectroscopy.

at%	C1s	O1s	Si2p
Sil-S-COONph 1	15.83	54.99	29.17
Sil-S-COONph 6	11.04	58.83	30.13
Sil-S-COONph 15	9.39	59.20	31.41

**Table 2:** Surface elemental composition of carboxylic acid-containing samples determined by X-ray photoelectron spectroscopy.

at%	C1s	O1s	Si2p
Sil-S-COOH 1	25.63	49.37	25.00
Sil-S-COOH 6	30.75	46.64	22.61
Sil-S-COOH 15	19.91	51.22	28.87

## Supplementary Materials

The following are available online at https://www.mdpi.com/article/10.3390/ijms222413289/s1.

## Abbreviations

The following abbreviations are used in this manuscript:

TEOS	tetraethyl orthosilicate
BNTES	butyronitrile triethoxysilane
TMSCl	trimethylsilyl chloride
Me	methyl groups
Et	ethyl units
