# Supplementary material for: Spherical Silica Functionalized by 2-Naphthalene Methanol Luminophores as a Phosphorescence Sensor
Source: Int J Mol Sci. 2021 Dec 10;22(24):13289. doi: 10.3390/ijms222413289 (PMC8703885; doi:10.3390/ijms222413289)
Supplement: Supplementary file 1 [file ijms-22-13289-s001.zip › ijms-1430368-supplementary/Supplementary Materials/Spherical_silica_functionalized_by_naphtalene_as_phosphorescence_indicator_IJMS_Rev2.pdf]

Supplementary Materials: Spherical silica functionalized by 2-naphthalenemethanol luminophores as a phosphorescence sensor

Magdalena Laskowska [1]*, Anna Nowak [1], Mateusz Dulski [2], Peter Weigl [3,4], Thomas Blochowicz [3], and Łukasz Laskowski [1]

1. Detailed procedures for the synthesis of Spherical silica functionalized by 2-naphthalenemethanol molecules

All reagents were purchased at the highest possible purity.

1.1. The synthesis of silica spheres with the diameter of 300 nm

The procedure provided here is an optimized synthesis route applied by Stöber and his team in [1]. It is based on hydrolysis and polycondensation of tetraetyl orthosilicate (TEOS – Sigma-Aldrich Chemie GmbH, Germany). The synthesis results in quite homogenous silica spheres with the diameter of 300 nm (299nm ± 11nm to be exact).

To obtain 0.6 g of spherical nanosilica the following reagents are required:

- deionized water: 10 cm^3;
- ethanol: 75 cm^3;
- TEOS: 5 cm^3;
- ammonia 25%: 4 cm^3.

The synthesis was performed in a 150 cm^3 polypropylene container with a cap. All procedures were carried out at ambient temperature. The process starts with mixing the solvents: water and ethanol under magnetic stirring. Next, TEOS was added and vigorously stirred for 10 min. After a clear solution was obtained, the ammonia was added as a catalyser of hydrolysis and polycondensation. The formation of spherical silica nanoparticles lasts for 10 hours under constant stirring. After the formation, the silica powder was recovered by centrifugation and washed a few times by ethanol and water taken from the excess of TEOS and ammonia. The powder was dried in vacuum at 120 °C for 24 h. The dry powder was stored in a tightly closed container in argon in order to avoid humidity (unwanted during functionalization).

1.2. The functionalization of silica spheres with carboxylic acid groups

The spherical silica can be functionalized by propyl carboxylic acid units by a grafting procedure. This can be done with a full substitution of the surface hydroxyl units by functional groups or with controlled distribution of acidic molecules on the surface achieved by the application of spacers. All the procedures were described in our earlier works [2–4]. Here, we provide example procedures in detail. https://www.overleaf.com/project/61950f1a5b8c301427a5b6a3

The presented procedure is for the controlled distribution with 1, 6 or 15 spacer units separating anchoring groups (N= 1, 6 or 15, respectively). The samples are denoted as SilS-COOH NX (where X denotes number of spacer units per single anchoring groups) for samples containing carboxyl groups. This ratio was set by changing the proportions between the precursors of functional units

Table S1. The amounts of precursors of anchoring units and spacer groups required to obtain the material with assumed number of spacer groups (N) per single anchor.

N	BNTES [g]	TEOS [g]
1	1.157	1.042
6	0.324	1.792
15	0.145	1.953

– 3-Cyanopropyltriethoxysilane (caled also butyronitrile triethoxysilane –BNTES, purchased from from Sigma-Aldrich Chemie GmbH, Germany) and the precursor of spacer units, TEOS. The mass of reagents, shown in Table S1, was calculated from the molecular weight of the corresponding constituents (TEOS – 208.33 g/mol, BNTES 231.36 g/mol).

The procedure consists of three steps. For the functionalization of 1.5 g of spherical silica, the following amounts of reagents are required:

Step 1:

- the spherical silica powder: 1.5 g;
- dichloromethane: 100 cm^3;
- TEOS: see: Table S1;
- BNTES see: Table S1;.

Step 2:

- the pre-functionalized spherical silica powder: 1.5 g;
- dichloromethane: 50 cm^3;
- chlorotrimethyl silane (ClTMS, Sigma-Aldrich Chemie GmbH, Germany): 3 cm^3.

Step 3:

- pre-functionalized silylated spherical silica powder: 1.5 g;
- concentrated hydrochloric acid: 25 cm^3;
- acetone: 20 cm^3
- deionized water: 5 cm^3.

Prior to the synthesis, the silica powder was thoroughly dried in vacuum at 120 °C for at least 24 hours. In the first step, the precursors of anchoring groups and spacers were dissolved in dichloromethane and mixed for at least two hours (until a clear solution was obtained). This was done on a magnetic stirrer in a round flask under the protective atmosphere of 1,1,1,2-Tetrafluoroethan, which is likely to remove any humidity. Next, dry spherical silica was added and the suspension was rigorously mixed under reflux for 24 hours. The pre-functionalized powder was recovered by centrifugation and washed several times with dichloromethane. After this procedure, the resulting powder was thoroughly dried in vacuum at the temperature of 120 °C for at least 24 hours and stored in a protective atmosphere.

In the next step (Step 2), the material was silylated in order to convert hydroxyl units into trimethyl silane groups (constituting spacers). For both types of materials, the procedure is the same. ClTMS was dissolved in dichloromethane, similarly to the previous step in, a round flask under a protective atmosphere. After obtaining clear solution (after approximately two hours), we added a dry pre-functionalized silica powder and stirred it under reflux for 24 h. The silylated powder was recovered by centrifugation and washed several times with dichloromethane. Next, the material was dried in vacuum at 120 °C for at least 24 hours.

In the last step, we hydrolyzed the precursors of anchoring units into carboxylic acid functional groups. The silica powder was dispersed into a mixture of HCl, acetone and DI water. The addition of acetone is necessary because the surface of silica spheres is strongly hydrophobic after silylation. The suspension was mixed under reflux for 24 h.The functionalized silica powder was recovered by centrifugation and washed several times by the mixture of DI water and acetone (till neutral pH was obtained). After drying in vacuum at 120 °C for at least 24 hours, the material is ready.

1.3. The functionalization of silica spheres with carboxylic acid groups by 2-naphthalene methanol

The material containing carboxyliuc acid anchores was subsequently activated by 2-naphthalene methanol luminophores. This procedure required following reagents:

- pre-functionalized silylated spherical silica powder: 1 g;

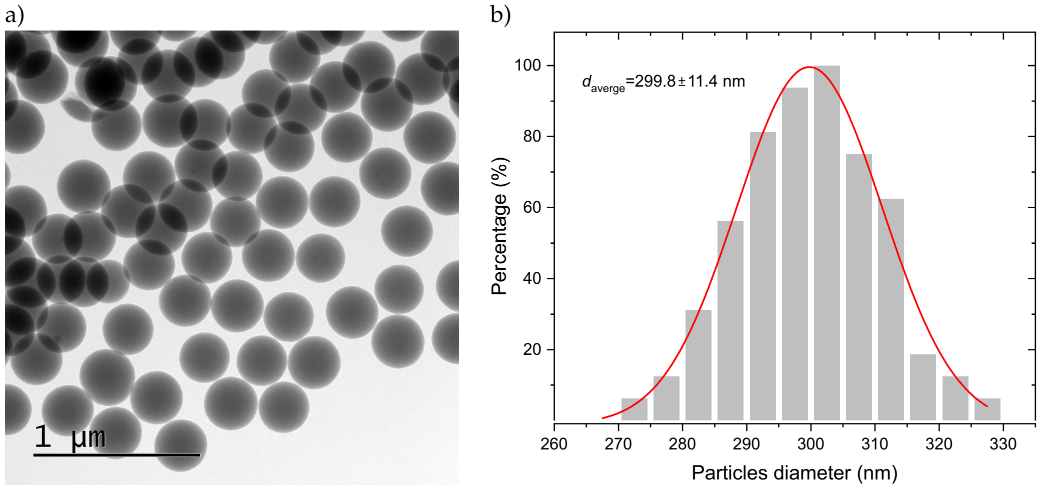

Figure S1. The distribution of the diameter of silica spheres obtained in the synthesis (b) along with the analyzed TEM image (a).

- benzine: 50 cm^3;
- ethanol anhydrous (99.9%): 50 cm^3
- 2-naphthalene methanol: 0.782 g.
- sulfuric acid 5M: a few drops for the adjusting of ph.

To do this, we dissolved 2-naphthalene methanol powder (purchased from from Sigma-Aldrich Chemie GmbH, Germany) in the 1:1 mixture of ethanol and benzine. As a catalyst we used sulfuric acid, being added to the solution, until ph 2 was reached. The suspension was mixed under reflux for 24 h. The powder was recovered by centrifugation and washed several times with the mixture of benzine and ethanol. The resulting materials were dried in vacuum for a few hours and stored under a protective atmosphere of 1,1,1,2-Tetrafluoroethan.

2. The distribution of the diameters of silica spheres

Just after synthesis, the native silica spheres has been analysed on the basis of Transmission Electron Microscopy (TEM) images. We used ImageJ for the processing of the images [5]. On this basis we plotted the distribution of the spheres diameter, ac can be seen in Figure S1, along with the analyzed image.

3. The assignment of individual Raman modes

Table S2. The assignments of individual Raman modes. ν - stretching modes, δ - deformational modes, Q_n - units characteristic for structurally disorder silica, * in oxygen correspond to unsaturated bonds.

Sil-S-COONph 1	Sil-S-COONph 6	Sil-S-COONph 15	Band assignment	Sil-S-COONph 1	Sil-S-COONph 6	Sil-S-COONph 15	Band assignment
Band position (cm^{-1})				Band position (cm^{-1})			
157	154	156	lattice modes, structural defects	157	156	156	lattice modes, structural defects
189	182	199		195	198	197	
227	229	235		233	237	237	
237	236	250		269	270	271	
311	306	321		316	316	315	
396	393	397		375	376	384	
403	404	406		403	404	404	

466	457	459	δ(Si-O*), δ(Si-O-Si)	447	442	451	δ(Si-O*), δ(Si-O-Si)
494	492	492		492	490	492	
546	543	542		544	543	546	
-	-	-		578	583	590	
599	605	601	δ(Si-O*)	-	-	-	-
681	685	693		-	-	-	
709	714	716		-	-	-	
783	774	775		-	-	-	
807	803	804	ν(Si-O*) in Q_n, Si vibration in an oxygen cage	803	803	803	ν(Si-O*) in Q_n
817	818	833		833	826	824	
-	-	-		880	882	884	
-	-	-		920	929	937	
-	-	-		-	-	-	
954	48	947	ν(Si-O*) in Q_n, δ(COO), skeletal C-C, δ(CH_x), x = 2,3	965	965	961	ν(Si-O*) in Q_n, δ(COO), skeletal C-C, νC=C, δ(CH_x), x = 2,3
980	981	983		983	985	982	
1023	1022	1022		1035	1022	1013	
1059	1049	1043		1055	1055	1055	
1083	1074	1074		1072	1075	1076	
1106	1097	1091		1091	1095	1095	
1179	1171	1179		-	-	-	
1205	1210	1218		-	-	-	
1245	1249	1251		1245	1246	1250	
1375	1371	1373		1368	1372	1370	
1402	1400	1401		1395	1398	1395	
1430	1430	1430		1432	1429	1431	
1459	1460	1467		1461	1460	1460	
1497	1467	1504		1491	1491	1487	
-	-	-		1522	1530	1582	
1609	1615	1609		1607	1611	1615	
1701	1701	1700	νC=O	1701	1701	1700	νC=O
-	-	-	ν(CH_x), x = 1,2,3	2731	2736	2728	ν(CH_x), x = 1,2,3
-	-	-		2816	2816	2816	
2843	2843	2844		2852	2852	2854	
2869	2874	2866		2883	2868	2868	
2906	2906	2906		2921	2924	2922	
2933	2934	2933		2937	2936	2935	
-	-	-		2953	2954	2943	
2973	2974	2973		2977	2977	2975	
3022	3022	3022		3021	3020	3020	
3072	3067	3077		3072	3069	3070	
3143	3144	3166		3121	3110	3101	
3252	3258	3272	ν(OH), water molecules, H-bonds	3245	3218	3315	ν(OH), water molecules, H-bonds
3441	3450	3452		3430	3452	3464	
3595	3605	3594		-	-	-	
3660	3665	3669		-	3615	3594	

Abbreviations

The following abbreviations were used in this manuscript:

TEOS tetraethyl ortosilicate
BNTES butyronitrile triethoxysilane / cyanopropyl triethoxysilane

References

1. Stöber, W.; Fink, A.; Bohn, E. Controlled growth of monodisperse silica spheres in the micron size range. *Journal of colloid and interface science* **1968**, *26*, 62–69.
2. Laskowska, M.; Oyama, M.; Kityk, I.; Marszalek, M.; Dulski, M.; Laskowski, L. Surface functionalization by silver-containing molecules with controlled distribution of functionalities. *Applied Surface Science* **2019**, *481*, 433–436.
3. Laskowski, L.; Kityk, I.; Konieczny, P.; Pastukh, O.; Schabikowski, M.; Laskowska, M. The Separation of the Mn12 Single-Molecule Magnets onto Spherical Silica Nanoparticles. *Nanomaterials* **2019**, *9*, 764.
4. Laskowska, M.; Pastukh, O.; Kuźma, D.; Laskowski, Ł. How to Control the Distribution of Anchored, Mn12–Stearate, Single-Molecule Magnets. *Nanomaterials* **2019**, *9*, 1730.
5. Schindelin, J.; Rueden, C.T.; Hiner, M.C.; Eliceiri, K.W. The ImageJ ecosystem: An open platform for biomedical image analysis. *Molecular reproduction and development* **2015**, *82*, 518–529.
