# Supplementary figures and images for: Spherical Silica Functionalized by 2-Naphthalene Methanol Luminophores as a Phosphorescence Sensor
Source: Int J Mol Sci. 2021 Dec 10;22(24):13289. doi: 10.3390/ijms222413289 (PMC8703885; doi:10.3390/ijms222413289)

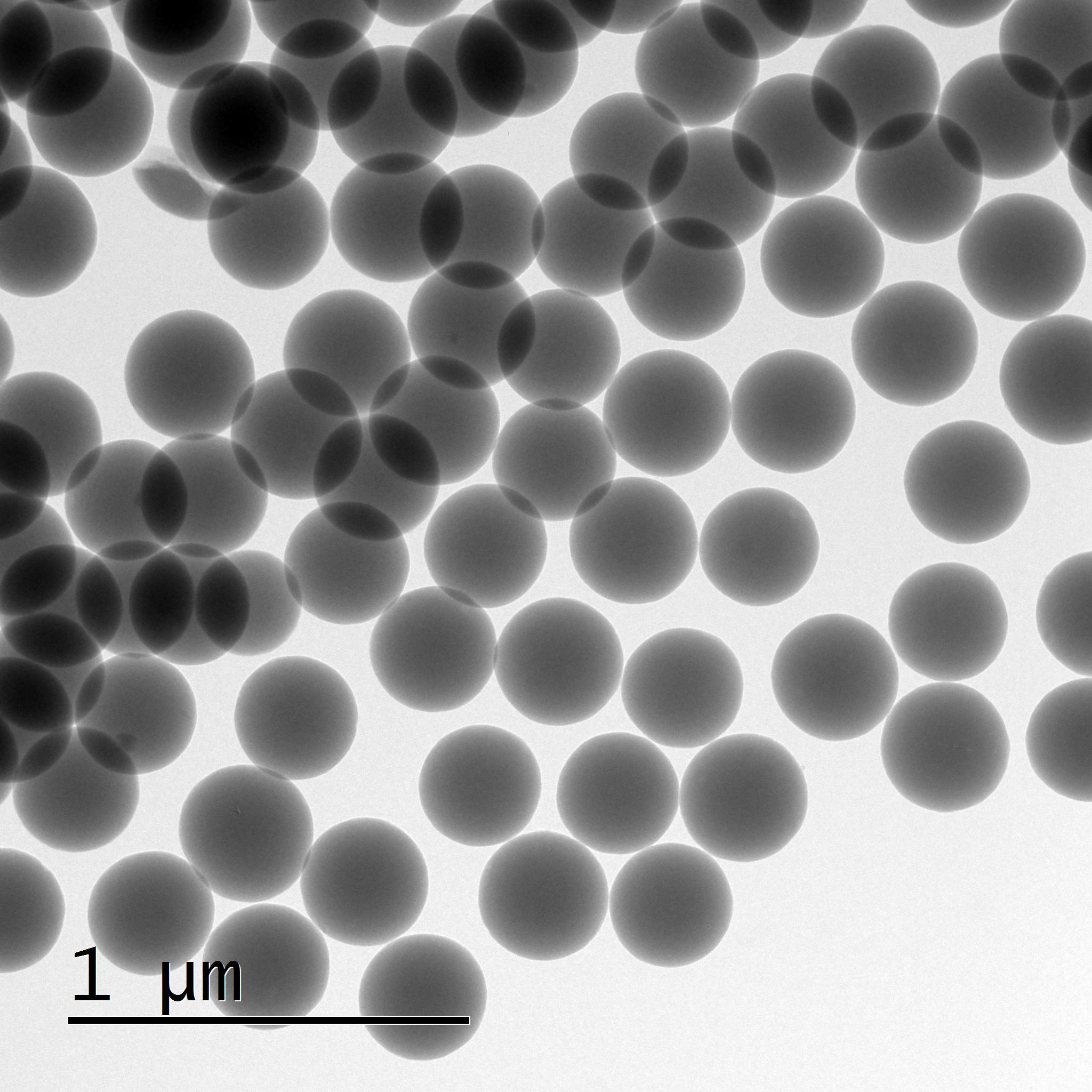

Supplement: Supplementary file 1 [file ijms-22-13289-s001.zip › ijms-1430368-supplementary/Supplementary Materials/figS1a.jpg]

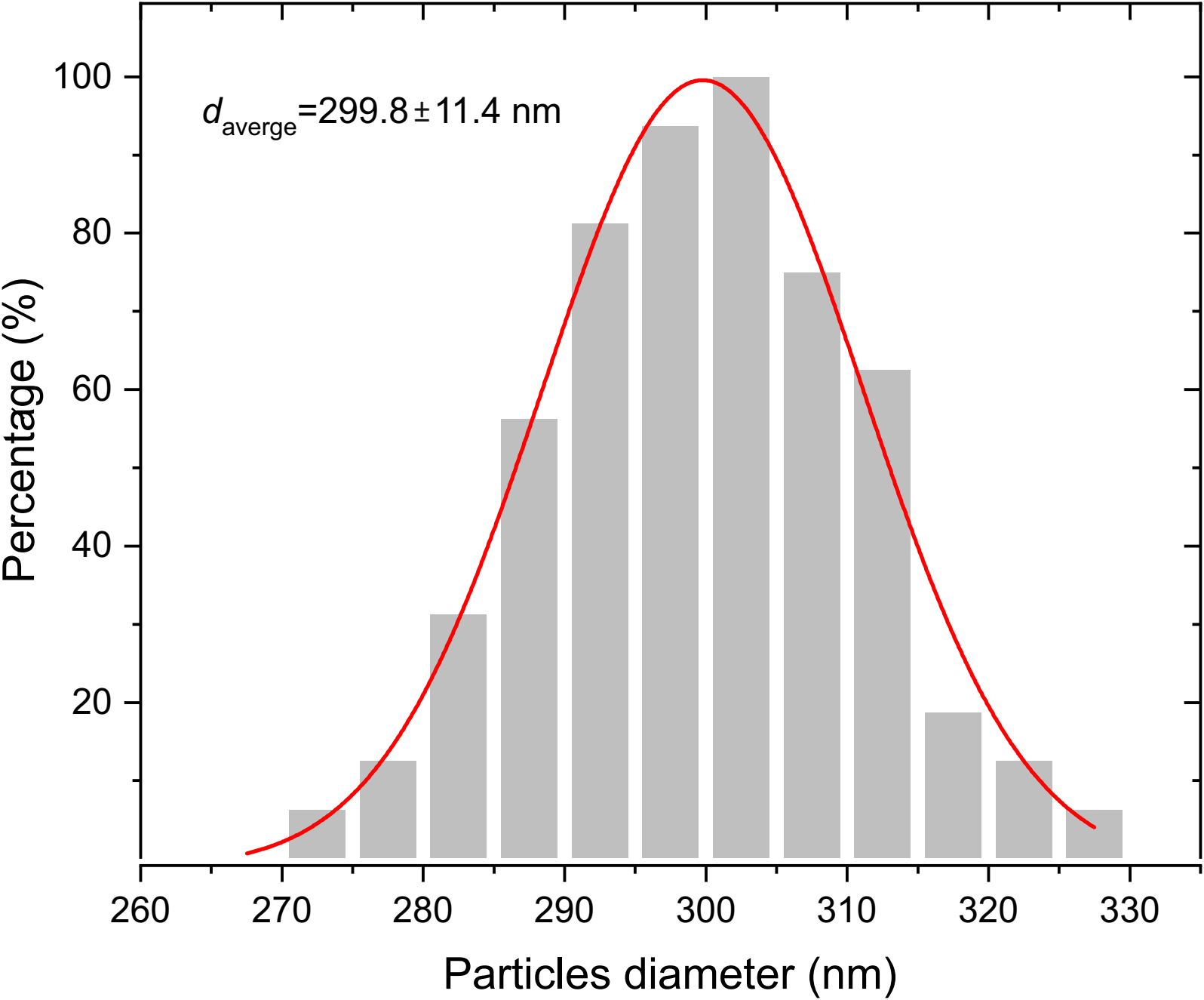

Supplement: Supplementary file 1 [file ijms-22-13289-s001.zip › ijms-1430368-supplementary/Supplementary Materials/figS1b.pdf]
